# Improving the accuracy of bone mineral density using a multisource CBCT

**DOI:** 10.1038/s41598-024-54529-4

**Published:** 2024-02-16

**Authors:** Yuanming Hu, Shuang Xu, Boyuan Li, Christina R. Inscoe, Donald A. Tyndall, Yueh Z. Lee, Jianping Lu, Otto Zhou

**Affiliations:** 1 Department of Physics and Astronomy, University of North Carolina at Chapel Hill, Chapel Hill, NC 27599 USA; 2 Department of Applied Physical Sciences, University of North Carolina at Chapel Hill, Chapel Hill, NC 27599 USA; 3 Department of Diagnostic Sciences, Adams School of Dentistry, University of North Carolina at Chapel Hill, Chapel Hill, NC 27599 USA; 4 Department of Radiology, University of North Carolina at Chapel Hill, Chapel Hill, NC 27599 USA

**Keywords:** Biomedical engineering, Imaging techniques and agents, Oral diseases, Imaging techniques

## Abstract

Multisource cone beam computed tomography CBCT (ms-CBCT) has been shown to overcome some of the inherent limitations of a conventional CBCT. The purpose of this study was to evaluate the accuracy of ms-CBCT for measuring the bone mineral density (BMD) of mandible and maxilla compared to the conventional CBCT. The values measured from a multi-detector CT (MDCT) were used as substitutes for the ground truth. An anthropomorphic adult skull and tissue equivalent head phantom and a homemade calibration phantom containing inserts with varying densities of calcium hydroxyapatite were imaged using the ms-CBCT, the ms-CBCT operating in the conventional single source CBCT mode, and two clinical CBCT scanners at similar imaging doses; and a clinical MDCT. The images of the anthropomorphic head phantom were reconstructed and registered, and the cortical and cancellous bones of the mandible and the maxilla were segmented. The measured CT Hounsfield Unit (HU) and Greyscale Value (GV) at multiple region-of-interests were converted to the BMD using scanner-specific calibration functions. The results from the various CBCT scanners were compared to that from the MDCT. Statistical analysis showed a significant improvement in the agreement between the ms-CBCT and MDCT compared to that between the CBCT and MDCT.

## Introduction

The use of dental implants to replace dentition and restore oral function and esthetics has revolutionized dental treatment. The stability and success of dental implants are determined by several factors, with bone quantity and quality as the most important determinant^1–3^. Bone mineral density (BMD) and bone microstructures are generally considered to be an excellent predictor of implant stability and osseointegration. Accurate measurement of the BMD prior to implant placement is highly recommended^4^. BMD is usually measured using radiological techniques, including dual-energy x-ray absorptiometry (DEXA)^5^ and computed tomography (CT)^6,7^. DEXA provides only an average areal density and is commonly used for osteoporosis screening. CT is an established method for acquiring the true volumetric mass density and differentiating cortical and trabecular bones^6,7^. However, because of the high cost, high radiation exposure to patients, and large footprint, medical CTs are not generally available in small dental clinics.

Cone beam CT (CBCT) generates 3D images with a high isotropic resolution at a reduced cost, lower radiation and smaller footprint compared to CT. It has become the 3D imaging device of choice at dental clinics^8,9^, and has been utilized to assess bone quality for pre-operative implant planning^8,10^. However, the CT Hounsfield Unit (HU) and consequently the BMD values derived from the current CBCT are inherently inaccurate^4,11,12^. To acquire volumetric images, CBCT uses a wide X-ray cone angle, resulting in strong scatter and cone beam artifacts compared to a multi-detector CT (MDCT). These lead to significant errors and spatial nonuniformity of the HU values^13^. Many commercial dental CBCT scanners only provide the image Grayscale Values (GVs) instead of the HU values.

Significant research has been devoted to improving the accuracy of CBCT for HU and BMD values. Techniques including anti-scattering grids, beam blockers, simulations, non-circular orbit, synthetic CT and machine learning have all been investigated^2,14–19^. Attempts have also been made to correlate the Greyscale values (GVs) from the CBCT with the CT numbers with limited success^12^. Recently, machine learning^20,21^ based techniques and dual-energy CBCT^22–24^ have demonstrated potential for improving the agreements with the MDCT measurements.

We recently demonstrated a multisource CBCT (ms-CBCT) technology^25–27^ that removes the root cause of the shortcomings of the current CBCT—the large X-ray beam cone angle—by replacing the single X-ray tube with a stack of narrowly collimated X-ray beams. The approach, investigated in the past by numerical and experimental simulations^26–29^, was realized for the first time using a carbon nanotube (CNT) field emission x-ray source array^30,31^. The ms-CBCT has been shown to reduce the scatter-primary ratio, essentially eliminate the cone beam artifacts, and improve the uniformity and accuracy of the CT HU values compared to a conventional single-source CBCT^26,27^. This study evaluated the accuracy of ms-CBCT for measuring the mandibular and maxilla bone density and compared its performance with the conventional CBCT.

## Materials and methods

### Multi-source CBCT

**Figure 1:**
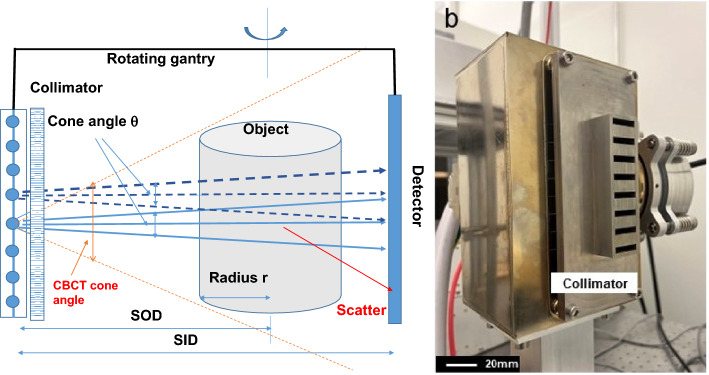
The design of the ms-CBCT. (**a**) A schematic of the ms-CBCT scanner. (**b**) A photo of the CNT x-ray source array and the external multisource collimator.

Figure 1 illustrates the design of the ms-CBCT. It consists of a customized CNT x-ray source array and a flat-panel detector (FPD) mounted on a rotating gantry. The source array (NuRay Technology Co., Ltd., Changzhou, China) contains eight focal spots with an inter-spot distance of 12 mm enclosed in a stainless-steel housing with a 1.7 mm thick Al window. The average focal spot size is 0.88 $\pm 0.05$ mm (width) by 1.10 mm$\pm 0.04$ (height) for the eight spots^32^. A multisource collimator confines the x-ray beam from each source to a narrow cone angle of 2.3° and a fan angle of 13.97° to illuminate only a small section of the object in the axial direction in each exposure. For comparison, a cone angle of 10° is required for a conventional CBCT configuration at the same system geometry and detector dimension. A CMOS flat panel detector using a CsI scintillator with an active area of 147.3 mm × 113.7 mm and a pixel size of 0.1 mm × 0.1 mm (Xineos-1511 from Teledyne DALSA in Waterloo, CA) was operated in the 2 × 2 binning mode. The source-to-axis distance (SAD) was 400 mm, and the source-to-detector distance (SDD) was 615 mm, similar to that of a commercial dental CBCT (CS9300, Carestream Dental). The FPD was laterally shifted to provide an effective field of view (FOV) of $187\mathrm{mm }\left({\text{weight}}\right)\times 100\mathrm{mm }\left({\text{height}}\right)$ at the rotational center to cover the entire maxillofacial region without truncation. The eight sources emitted X-rays sequentially in a cycle. 360 projection images were collected from each source in a 360° gantry rotation, resulting in a total of 2880 exposures per rotation by the eight sources.

The projection images were processed by an Adjacent Scattering Ratio Subtraction (ASRS) algorithm to remove the residual scatter from primarily in-plane scattering^26^. Volumetric 3D reconstruction was conducted by the 3D Simultaneous Iterative Reconstruction Technique (SIRT) algorithm using the ASTRA Toolbox^33,34^. Noise reduction was achieved by a total variation (TV) algorithm using the Tigre Toolbox^35^. All reconstructions had their HU calibrated in accordance with the testing instructions for the Gammex CT ACR 464 phantom^36^.

## Phantoms

**Figure 2:**
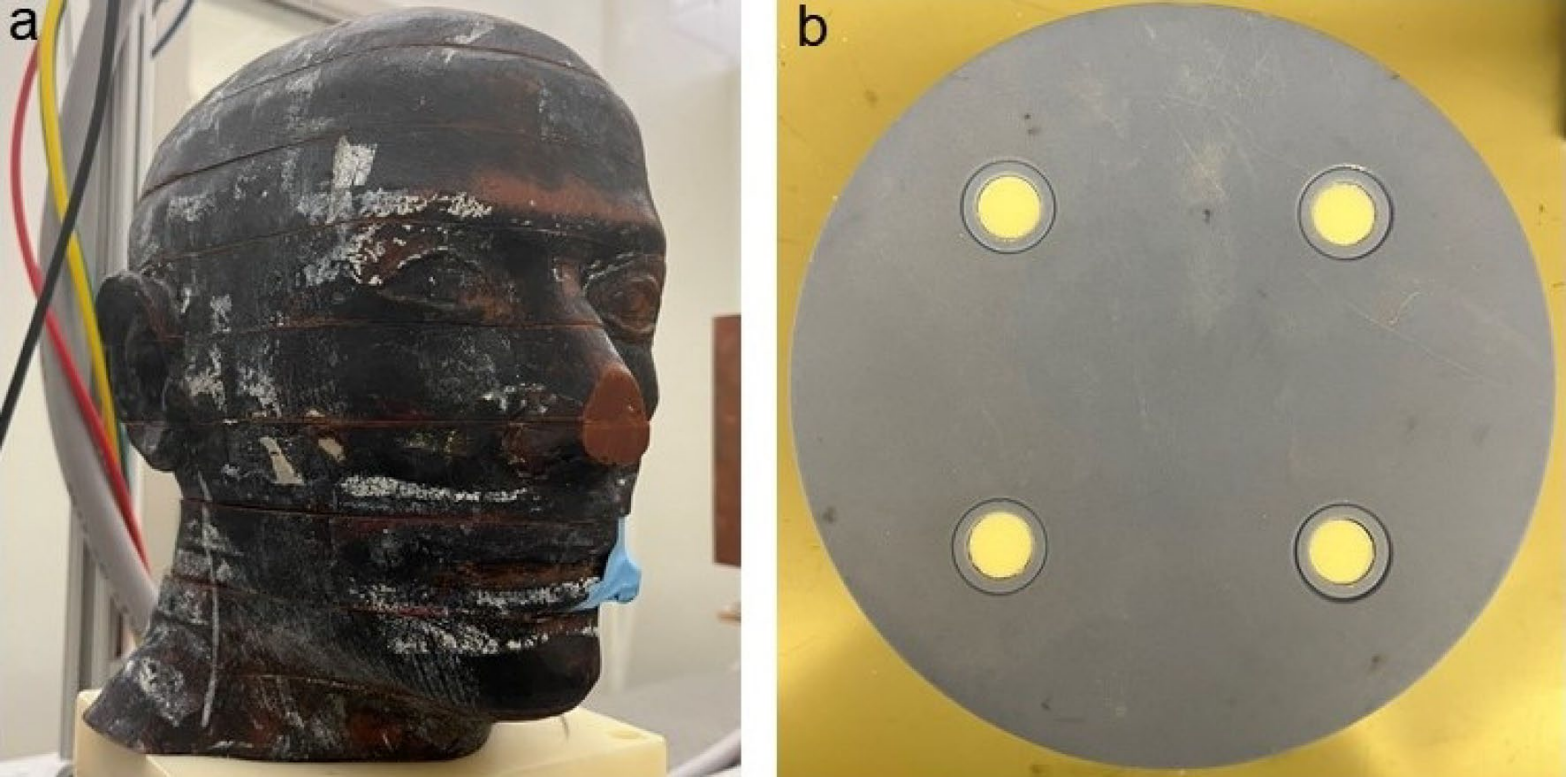
(**a**) The anthropomorphic RANDO phantom consisting of multiple slabs with finite air gaps in between; (**b**) The home-made calibration phantom consisting of a 16 cm diameter SolidWater cylinder with 4 wells hosting removable inserts.

An anthropomorphic adult skull and tissue-equivalent head phantom (RANDO, Nuclear Associates, Hicksville, NY) and a homemade calibration phantom, comprised of a 16 cm diameter water equivalent plastic cylinder (SolidWater, Sun Nuclear Co, Melbourne FL) with four 1.3 cm diameter wells, as shown in Fig. 2, were used. A total of 12 inserts were made for the calibration phantom, each composed of a compressed mixture of hydroxyapatite (HAp) (Acros Organics, Fisher Scientific, Pittsburgh, PA) and polyethylene (PE) powders (Alfa Aesar, Ward Hill) in different proportions, as listed in Table 1. Powders with specific mass ratios of HAp and PE were thoroughly mixed and then compressed into cylindrical inserts, each with a diameter of 13 mm. Following the pressing process, the total mass and height of each insert were measured. The corresponding BMD was calculated as: $BMD=\frac{{M}_{HAp}}{{V}_{total}}$, where M_HAp_ represents the weight o the HAp and V_total_ volume of the insert.

**Table 1:** Composition and density of the inserts used for BMD calibration.

Inserts		M_total_^b^ (mg)	M_HAp_^b^ (mg)	V_total_^c^ (mm^3^)	BMD^d^ (mg/cm^3^)
1	95% HAp^a^; 5% PE^a^	1695.2	1610.44	942.40	1709
2	90% HAp; 10% PE	1592.7	1433.43	939.74	1525
3	85% HAp; 15% PE	1598.3	1358.56	991.51	1370
4	80% HAp; 20% PE	1478.1	1182.48	922.49	1282
5	75% HAp; 25% PE	1207.8	905.85	836.21	1083
6	65% HAp; 35% PE	1591.4	1034.41	1189.28	870
7	55% HAp; 45% PE	1669	917.95	1329.98	690
8	45% HAp; 55% PE	1460.4	657.18	1260.96	521
9	35% HAp; 65% PE	1679.7	587.90	1533.06	383
10	25% HAp; 75% PE	1144.8	286.20	1157.43	247
11	15% HAp; 85% PE	1417.4	212.61	1470.67	145
12	5% HAp; 95% PE	1453.5	72.68	1588.81	46

^a^*HAp* hydroxyapatite, *PE* polyethylene. The percentage number is the weight percentage of each component.

^b^*M*_*total*_ total mass of the inserts, *M*_*HAp*_ the product of the total mass and the corresponding HAp mass ratio in the specific inserts.

^c^*V*_*total*_ calculated volume of the inserts.

^d^*BMD* bone mineral density.

## Image acquisition

The phantom images were acquired using five scanners: the ms-CBCT, the ms-CBCT operating at the conventional CBCT configuration using only one source at a wide cone angle to cover the entire FOV (referring to as “CBCT-1”), NT5G (NewTom, Italy) clinical CBCT scanner (referring to as “CBCT-N”), 3D Accuitomo 170 (J. Morita, Japan) clinical CBCT scanner (referring to as “CBCT-M”), and SOMATOM Force (Siemens, Germany) clinical MDCT. The standard protocols in our institution for adult patients were used for the clinical scanners. The CBCT-N was operated at manufacturer suggested 110 kVp and CBCT-M in the continuous mode at manufacturer suggested 90 kV. The ms-CBCT, CBCT-1, and the MDCT were operated at both 90 kVp and 110 kVp.

For the ms-CBCT and the CBCT-1, the dose area product (DAP) values were computed in accordance with the equation below:

1 $$DAP=D\times\Delta {t}_{exp}\times A\times {N}_{view}\times {N}_{source}$$

where $D$ represents the experimentally measured dose rate at the detector, $\Delta {t}_{exp}$ is the exposure time per source per view, $A$ the area of the detector segment illuminated by the primary photons for each exposure (the detector width × full-width half maximum of the primary beam in the axial direction), ${N}_{source}$ the total number of sources, which is 8 for the ms-CBCT and 1 for CBCT-1, and ${N}_{view}$ the number of projection views per source, which is 360 over 360° degrees. The dose rates for ms-CBCT and CBCT-1 were previously measured at $86.6\upmu Gy/mAs$ and $156.0\upmu Gy/mAs$ for 90 kVp and 110 kVp, respectively^26^. The value of $A$ was $147.1 {\text{mm}} \left(width\right)\times 32.5 {\text{mm}}\left(height\right)$ for each beam for the ms-CBCT and $147.1 {\text{mm}} \left(width\right)\times 113.7 {\text{mm}}\left(height\right)$, the entire detector area, for the CBCT-1. The detailed imaging protocols are listed in Table 2.

**Table 2:** Imaging protocols for the MDCT and the CBCT devices used in this study.

Filtration	MDCT		ms-CBCT		CBCT-1		CBCT-M	CBCT-N
	0.6 mm Sn		1.7 mm Al + 0.3 mm Cu		1.7 mm Al + 0.3 mm Cu		5.5 mm Al	6.0 mm Al
Tube voltage(kVp)	90	110	90	110	90	110	90	110
Tube current(mA)	72	72	15	11	15	11	6	8
Exposure Time per view per source (ms)			6.5	5.0	6.5	5.0		
Total Exposure Time (s)	1.00	1.00	18.72	14.40	2.34	1.80	17.50	10.00
Exposure (mAs)	90.00	90.00	35.10	19.80	35.10	19.80	105.00	80.00
FOV (cm^2^)			$18.7{\text{x}}10.0$		$18.7\times 7.4$		$17\times 12$	$18\times 16$
DAP (dGy*cm^2^)			11.63	11.81	5.08	5.17	30.7*	15.25

*Continuous exposure.

## Pre-processing

Due to variations in the scanner geometry and phantom placement, the reconstructed CBCT images from the 4 CBCT scanners were first matched with the MDCT image using a software called 3D Slicer^37^. The volumes containing the mandible and maxilla were cropped and registered as a rigid body with the MDCT images. Binary masks were applied to separate the volume of interests (cortical and cancellous regions of the mandible and the maxilla) from the rest of the imaging volume. The binary masks for the cortical bones were generated by threshold segmentation, and the non-target bones were removed manually. The low HU/GV values of the cancellous bones prevented application of the threshold segmentation method. Instead, the mask was generated by manually filling the space and subtracting the cortical bone region. The final masks were generated by selecting the overlapping regions from the binary masks of the images from the four scanners at each x-ray tube energy. Data sampling was performed by selecting the sagittal slices from the overlapped regions as the region of interests (ROIs) with a slice thickness of $0.46\mathrm{ mm}$. For the mandible, only the lower section was used to avoid contamination from the roots of the teeth. This yielded 131 ROIs each for both the cortical and cancellous bones, and 228 ROIs at 110 kVp and 207 ROIs at 90 kVp) for the maxilla. For the calibration phantom, binary masks were generated to encompass the entire volume of the inserts. Subsequently, the CBCT images were resampled to match the voxel size of the MDCT, which is $0.36*0.36*1.5 {{\text{mm}}}^{3}$.

## Data analysis

The correlation between the HU/GV value and the corresponding BMDs was established for each scanner by linear regression of the experimentally measured HU/GV values with the known BMD values of the inserts in the calibration phantom. The scanner-specific relation was then used to transform the HU/GV values of the RANDO phantom to the BMDs. The results from each CBCT scanner were compared to the values from the MDCT for the same ROI, which was used as the substitute for the ground truth since the actual composition and density of the RANDO phantom are unknown. Both line regression and the Bland–Altman (BA)^38^ analysis were used to evaluate the agreement and correlation between BMDs derived from each CBCT scanner and the MDCT.

## Results

### Reconstructed images and segmentations

**Figure 3:**
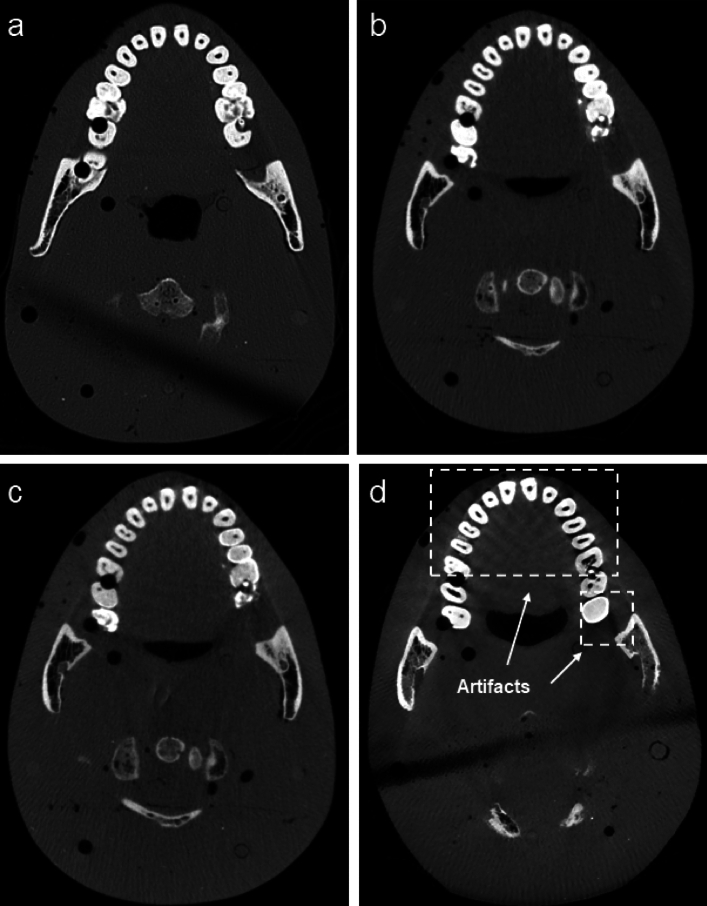
Axial images of the RANDO phantom from the MDCT (**a**) and CBCTs (**b**–**d**) at 110 kVp (window level: 600 HU, window width: 2800 HU). (**b**) ms-CBCT, (**c**) CBCT-1, (**d**) CBCT-N. The dark band is caused by the air gap between the multiple slabs of the RANDO phantom.

**Figure 4:**
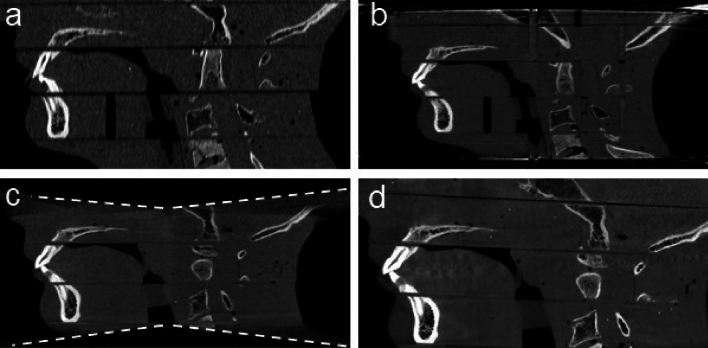
Sagittal images of the RANDO phantom from MDCT (**a**) and three CBCT scanners (**b**–**d**) at 110 kVp (window level: 600 HU, window width: 2800 HU). (**b**) ms-CBCT, (**c**) CBCT-1, (**d**) CBCT-N. The dark horizontal lines are from the air gap between the individual slabs of the RANDO phantom.

Figures 3 and 4 show the axial and sagittal slices of the reconstructed images of the RANDO phantom from the MDCT and 3 CBCT scanners at 110 kVp and displayed at the same window levels. High quality images were obtained from the ms-CBCT. Compared to the images from the two conventional CBCT scanners, the ms-CBCT images contain fewer artifacts around the dental structures, as depicted in Fig. 3b,d. Compared to the single source CBCT-1, which has the conventional CBCT configuration and the same FPD and magnification factor, the ms-CBCT has an expanded field of view in the axial direction, as demonstrated by the differences in the axial coverage be in Fig. 4b,c.

**Figure 5:**
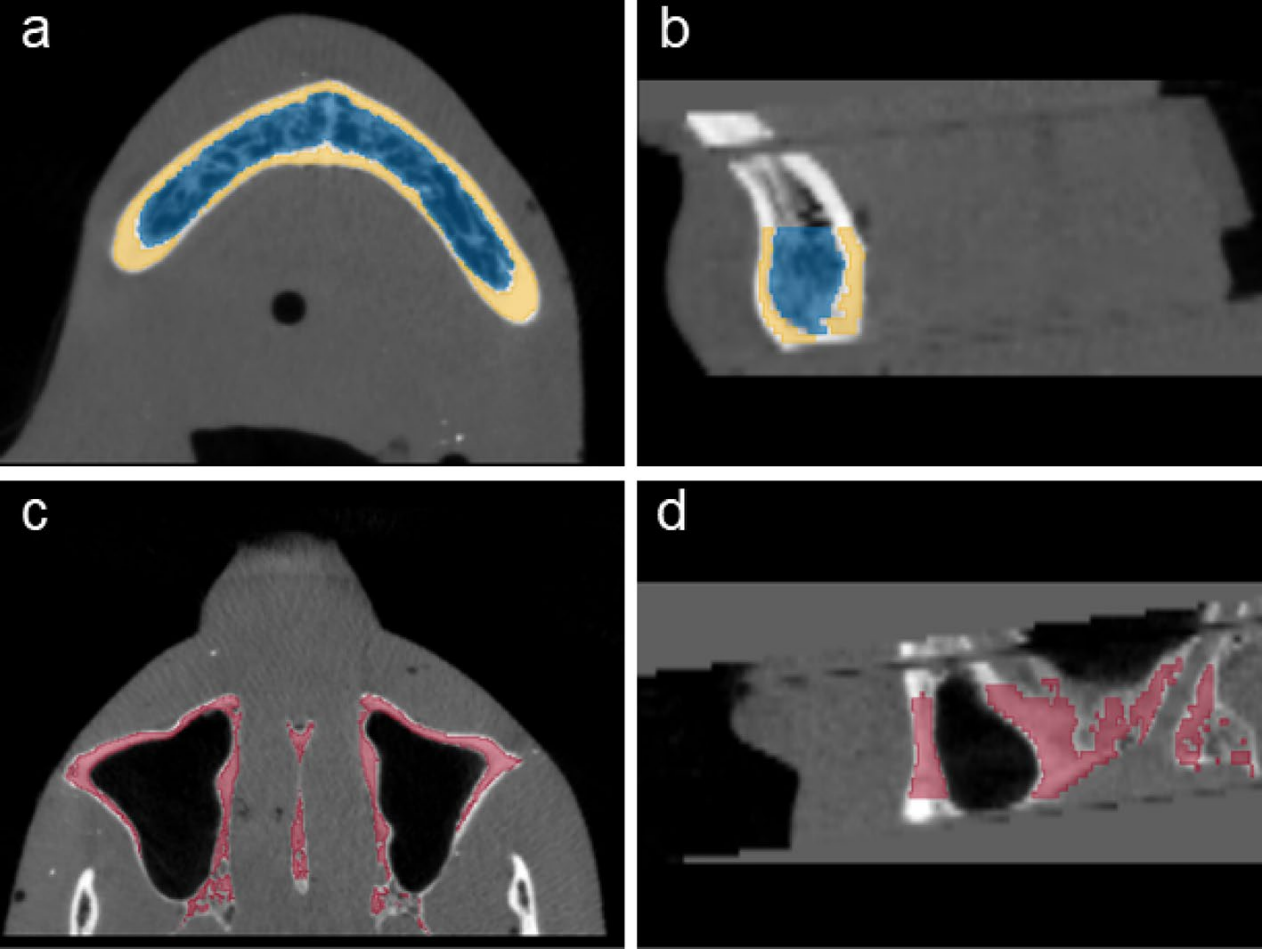
The RANDO phantom imaged using the ms-CBCT, after registration with the image from the MDCT and segmentation. (**a**,**b**) Segmentation of mandible , with yellow indicating the cortical bone and blue representing the cancellous bone. (**c**,**d**) Segmentation of maxilla.

Figure 5 shows the reconstructed slices of the RANDO phantom imaged using the ms-CBCT at 110 kVp, after registration with the image from the MDCT at the same x-ray energy and segmentation. Figure 5a,b are respectively the axial and sagittal slices showing segmented cortical (yellow) and cancellous (blue) bones of the mandible. As described in the method section, only the lower section of the mandibular bones was selected as the ROIs for this study to avoid contamination from the tooth root. Figure 5c,d are the axial and sagittal slices showing the segmented maxilla. Similarly, due to the presence of artifacts at the top edge of FOV and the tooth root at the bottom region of the maxilla, only the central section was selected.

## Bone mineral density

Table 3 shows the correlation between the HU/GV and the known BMD for all five CBCT/CT scanners used in this study. The results were obtained by linear regression of the HU/GV from the reconstructed CT images and the known BMD of the HAp inserts of the contrast phantom. A linear relation with the coefficient of determination (R^2^) values larger than 0.99 was obtained for all scanners. The averaged HU/GV values measured from the ROIs of the segmented mandibular and maxilla bones of the RANDO phantom were converted to the BMDs using these scanner-specific equations.

**Table 3:** Relation between the measured HU/GV and the known BMD values from the calibration.

Tube voltage	90 kVp		110 kVp	
Devices	Linear regression equation (y = ax + b) *	R^2^	Linear regression equation (y = ax + b)	R^2^
MDCT	y = 0.77x + 147.03	0.9970	y = 0.90x + 167.98	0.9968
ms-CBCT	y = 0.94x + 178.39	0.9962	y = 1.08x + 201.39	0.9942
CBCT-1	y = 1.06x + 153.77	0.9954	y = 1.22x + 165.06	0.9954
CBCT-M	y = 0.97x + 56.78	0.9924		
CBCT-N			y = 1.08x + 28.21	0.9909

*y represents the BMD and x represents the HU/GV.

**Figure 6:**
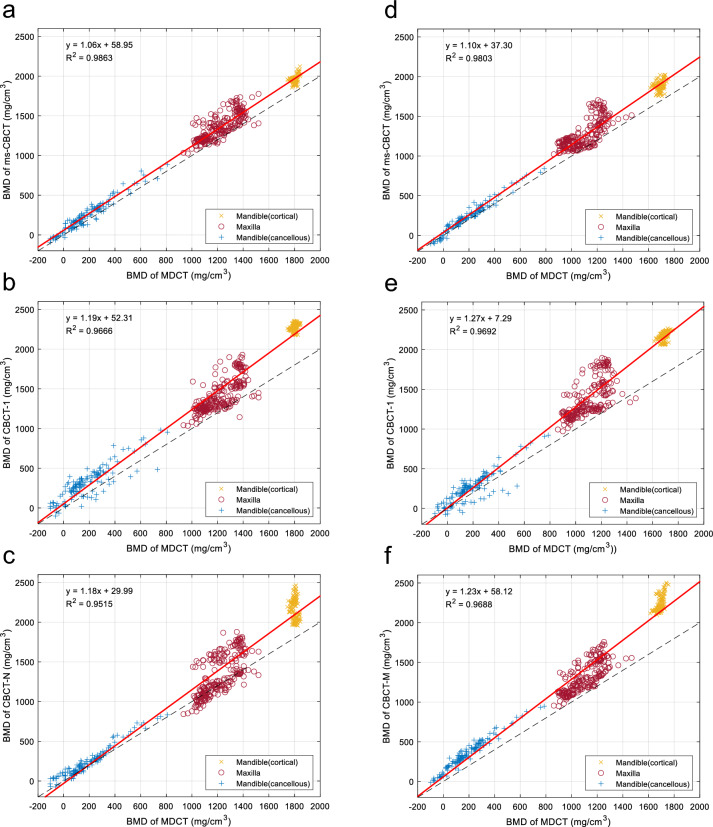
The BMD values of the mandible and maxilla derived from the CBCT versus the values from the MDCT at 110 kV (**a**–**c**) and 90 kV (**d**–**f**). (**a**) ms-CBCT vs MDCT at 110 kVp; (**b**) CBCT-1 vs MDCT at 110 kVp; (**c**) CBCT-B vs MDCT at 110 kVp; (**d**) ms-CBCT vs MDCT at 90 kVp; (**e**) CBCT-1 vs MDCT at 90 kVp; (**f**) CBCT-M vs MDCT at 90 kVp. The red solid line represents the linear regression fit for the data, while the black dashed line denotes the line of identity (y = x), serving as a reference.

The BMDs for each CBCT scanner (ms-CBCT, CBCT-1 and two clinical CBCTs) were plotted against the values derived from the MDCT for the same ROIs and are shown in Fig. 6, along with the equality line. Each data set was fitted with a linear equation of $y = ax + b$, where $y$ is the BMD value from the CBCT, $x$ is the BMD value from the MDCT, and $a$ and $b$ are the fitting parameters. The results are shown as the red solid lines in Fig. 6. Comparing to the MDCT, the ms-CBCT overestimated the BMD values by 6% at 110 kVp and 10% at 90 kVp. This a substantial reduction of the discrepancy between a conventional CBCT and the MDCT. As shown, the deviations between the conventional CBCT scanners (CBCT-1, CBCT-N and CBCT-M) and the MDCT are 18–19% at 110 kVp and 23–27% at 90 kVp.

**Figure 7:**
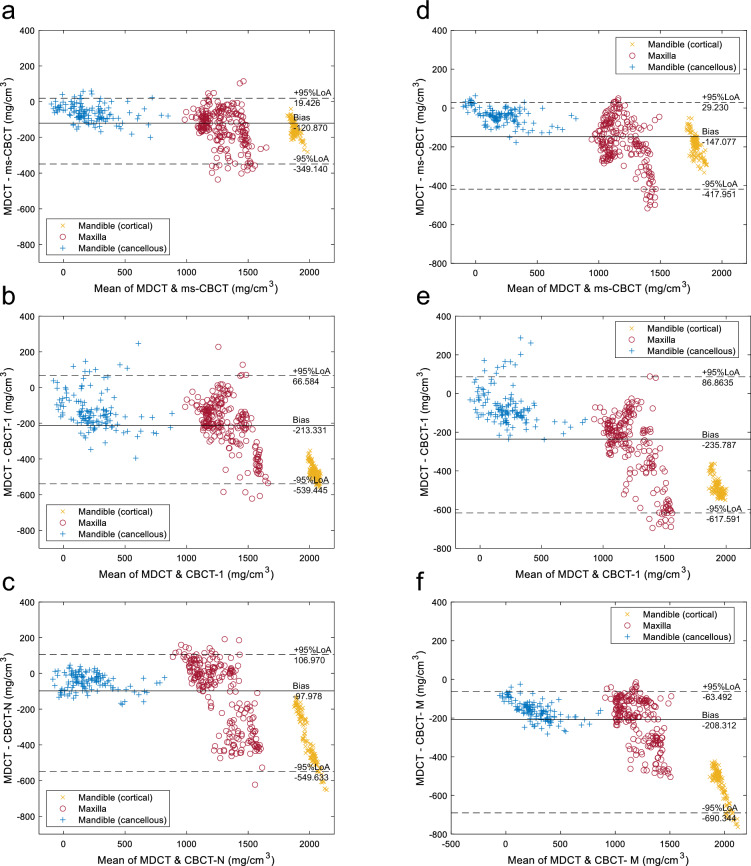
The Bland–Altman plots between the MDCT and ms-CBCT, CBCT-1, CBCT-N, and CBCT-M images. (**a**–**c**) CBCT images at the mandible and maxilla under the tube voltage of 110 kVp, (**d**–**f**) CBCT images at the mandible and maxilla under t the tube voltage of 90 kVp. The solid line represents the median difference (bias), and the dashed lines represent the upper (+ 95%LoA) and lower (− 95%LoA) 95% limits of agreement (LoA), which were estimated using the 2.5th and 97.5th percentiles due to non-normality of the data.

The BMD values from the various ROIs in the mandible and maxilla do not strictly follow a normal distribution, which is required for the normal Bland–Altman plot. A modified version of the Bland–Altman plot based on non-parametric methods was generated^38,39^. In this version, the limits of agreement (LoA) were estimated using the 2.5th and 97.5th percentiles, and the average bias was calculated as the median of the differences. The data were plotted in Fig. 7 as the difference versus the mean of the BMD values derived from the CBCT and the MDCT. The averaged bias is shown as a solid line, and the 95% LoA as the dash lines.

At 90 kVp, the bias for the ms-CBCT was − 147.077 mg/cm^3^ with the 95% LoA width of 447.181 mg/cm^3^. In comparison, for the two conventional CBCT scanners, CBCT-1 and CBCT-M, the biases were − 235.787 mg/cm^3^ and − 208.312 mg/cm^3^ with the LoA widths of 758.455 mg/cm^3^ and 753.836 mg/cm^3^, respectively. The width of the 95% LoA for the ms-CBCT is significantly narrower than that for the conventional CBCT. At 110 kVp, the bias of the ms-CBCT was − 120.870 mg/cm^3^, with the LoA width of 368.566 mg/cm^3^. In comparison, the conventional CBCT-1 and CBCT-N show biases of − 213.331 mg/cm^3^ and − 97.978 mg/cm^3^ and the LoA width of 606.029 mg/cm^3^ and 659.633 mg/cm^3^, respectively. The results are summarized in Table 4.

**Table 4:** Results from the Bland–Altman type analysis of the difference between the CBCTs and MDCT for BMD measurements.

	Mandible (cortical & cancellous) and Maxilla	
	Bias (mg/cm^3^)	95% LoA (mg/cm^3^)
110 kVp Group		
ms-CBCT	− 120.870	368.566
CBCT-1	− 213.331	606.029
CBCT-N	− 97.978	659.633
90 kVp Group		
ms-CBCT	− 147.077	447.181
CBCT-1	− 235.787	758.455
CBCT-M	− 208.312	753.836

## Discussion

In this study, the newly developed multisource CBCT was evaluated for its accuracy in determining the BMD of the mandible and maxilla compared to the current generation of clinical CBCT scanners. Because the composition and mass densities of the anthropomorphic head phantom are unknown, experimentally measured BMD values from a clinical MDCT were used as the reference. The clinical MDCT, which is periodically calibrated for quantitative measurements, is known to be significantly more accurate than the CBCT and is commonly used as a substitute for the ground truth for this type of studies^20–24^. The linear regression results show that the conventional CBCT scanners overestimate the BMD values by 23–27% at 90 kVp and 18–19% at 110 kVp. The large deviations observed are consistent with the results from previous studies of CBCT^20,21,40^. In comparison, the discrepancy between the ms-CBCT and the MDCT was reduced substantially to 10% at 90 kVp and 6% at 110 kVp. Similarly, Bland–Altman type analysis show the limits of agreement width between the CBCT and MDCT was reduced from 753.8–758.4 mg/cm^3^ at 90 kVp and 606.0–659.6 mg/cm^3^ at 110 kV for the conventional CBCT to respectively 447.2 mg/cm^3^ and 368.6 mg/cm^3^ for the ms-CBCT.

Previous efforts on improving the accuracies of HU and BMD values primarily relied on software based post processing methods such as empirically collaborating the CBCT Greyscale Value with the HU and machine learning. As summarized in several reviews^11,12,41^, GVs derived from CBCT are unreliable and vary significantly with the imaging parameters used, limiting the applicability of the correlation approach. A recent study applied a hybrid deep-learning model to the CBCT images of dry human skull phantoms similar to the one used in this study^20^. The limits of agreement width with the MDCT in the Bland–Altman analysis was narrowed to between 770 mg/cm^3^ and 990 mg/cm^3^. In comparison, the improvement was achieved in ms-CBCT by directly addressing the root cause limitation of the conventional CBCT design, which is the large x-ray cone angle used for volumetric imaging. Without applying any post processing, a significantly smaller variation (368.6 mg/cm^3^ at 110 kV) was obtained. The scanner provides better quality raw data compared to a conventional CBCT. The software based techniques developed for the conventional CBCT such as machine learning can be utilized to further enhance the results.

The ms-CBCT is essentially multiple axial CT stacked in the axial direction. In the present design, the cone angle of each individual beam is 2.3°, compared to ~ 10° for a conventional CBCT at the same system geometry, as in the case of the CBCT-1. It has been demonstrated previously^25–29^ that the small cone angle reduces scatter and increases the uniformity and accuracy of the CT HU values. Studies have also shown the dependence of the CBCT HU accuracy on the FOV used for imaging acquisition, for the same reason. The increased accuracy of the BMD observed in this study is consistent with these findings.

In this study, the standard clinical imaging protocols were used for the two clinical CBCT scanners. The DAP used for the ms-CBCT is comparable to the value of the clinical CBCT-N. Both used pulsed X-ray radiations. The DAP for the CBCT-M is higher, presumably because the scanner uses a continuous X-ray radiation. The axial field of view of the ms-CBCT is slightly smaller than that of the CBCT-M and CBCT-N (10 cm vs 12 cm and 16 cm respectively). This small variation does not have a significant effect on the large difference in the performances observed between the scanners. In addition, for control purpose, CBCT-1, which has the same system geometry and exposure conditions as the ms-CBCT except only one source is used in the conventional CBCT configuration was included in this study. The results from the CBCT-1 are similar to those from the clinical CBCT-N and CBCT-M, supporting the significantly improved performance from the multi-source design.

Becauae of the large errors of the current clinical CBCT in measuring the HU and BMD values, clinicians often need to rely on experience rather than quantitative measures to assess the bone quality today. Increased accuracy will potentially help clinicans assess the bone qualiy, improve the reliability of implant site selection and predict the stability of implants. The results of this study, in addition to the improved soft tissue contrast resolution and uniformity and accuracy of the CT HU values reported previously^26,27^, demonstrate that the ms-CBCT design offer significant advantages over the current generation of CBCT.

## Conclusion

This study demonstrates that the new multisource CBCT significantly improves the accuracy of bone mineral density measurement compared to the current generation of clinical CBCT systems without increasing the imaging dose. The device provides 3D volumetric imaging capability at the quality and accuracy similar to that of the MDCT using the imaging dose and footprint of a CBCT. It can potentially provide quantitative preoperative assessment of the bone quality for dental implants.

## Data Availability

All data that support the findings of this study are included within the article.

## Abbreviations

CBCT-1: The ms-CBCT operating in the conventional CBCT configuration, with a single source at a wide cone angle to cover the entire FOV
CBCT-M: A clinical CBCT scanner, model 3D Accuitomo 170 by J. Morita, Japan
CBCT-N: A clinical CBCT scanner, model NT5G by NewTom, Italy
DEXA: Dual-Energy X-ray Absorptiometry
GV: Greyscale Value
HU: CT Hounsfield Unit
MDCT: Multi-Detector CT
ms-CBCT: Multisource Cone Beam Computed Tomography

## References

[1] Merheb J (2010). Relationship between cortical bone thickness or computerized tomography-derived bone density values and implant stability. Clin. Oral Implant Res..

[2] Ribeiro-Rotta RF, de Oliveira RCG, Dias DR, Lindh C, Leles CR (2014). Bone tissue microarchitectural characteristics at dental implant sites part 2: Correlation with bone classification and primary stability. Clin. Oral Implant Res..

[3] Rues S (2021). Effect of bone quality and quantity on the primary stability of dental implants in a simulated bicortical placement. Clin. Oral Invest..

[4] Miguel-Sánchez A, Vilaplana-Vivo J, Vilaplana-Vivo C, Vilaplana-Gómez JÁ, Camacho-Alonso F (2015). Accuracy of quantitative computed tomography bone mineral density measurements in mandibles: A cadaveric study. Clin. Implant Dent. Relat. Res..

[5] Khalatbari H, Binkovitz LA, Parisi MT (2021). Dual-energy X-ray absorptiometry bone densitometry in pediatrics: A practical review and update. Pediatr. Radiol..

[6] Nickoloff EL, Feldman F, Atherton JV (1988). Bone mineral assessment: New dual-energy CT approach. Radiology.

[7] Booz C (2020). Diagnostic accuracy of quantitative dual-energy CT-based bone mineral density assessment in comparison to Hounsfield unit measurements using dual x-ray absorptiometry as standard of reference. Eur. J. Radiol..

[8] Scarfe, W. C. & Angelopoulos, C. *Maxillofacial Cone Beam Computed Tomography* (Springer, 2018).

[9] Gaêta-Araujo H (2020). Cone beam computed tomography in dentomaxillofacial radiology: A two-decade overview. Dentomaxillofac. Radiol..

[10] Jacobs R, Salmon B, Codari M, Hassan B, Bornstein MM (2018). Cone beam computed tomography in implant dentistry: Recommendations for clinical use. BMC Oral Health.

[11] Molteni R (2013). Prospects and challenges of rendering tissue density in Hounsfield units for cone beam computed tomography. Oral Surg. Oral Med. Oral Pathol. Oral Radiol..

[12] Pauwels R, Jacobs R, Singer SR, Mupparapu M (2015). CBCT-based bone quality assessment: Are Hounsfield units applicable?. Dentomaxillofac. Radiol..

[13] Siewerdsen JH, Jaffray DA (2001). Cone-beam computed tomography with a flat-panel imager: Magnitude and effects of x-ray scatter. Med. Phys..

[14] Stankovic U, Ploeger LS, van Herk M, Sonke JJ (2017). Optimal combination of anti-scatter grids and software correction for CBCT imaging. Med. Phys..

[15] Lee H, Lee J (2019). A deep learning-based scatter correction of simulated x-ray images. Electronics.

[16] Liu Y (2020). CBCT-based synthetic CT generation using deep-attention cycleGAN for pancreatic adaptive radiotherapy. Med. Phys..

[17] Zhang Y (2021). Improving CBCT quality to CT level using deep learning with generative adversarial network. Med. Phys..

[18] Hatamikia S (2022). Source-detector trajectory optimization in cone-beam computed tomography: A comprehensive review on today’s state-of-the-art. Phys. Med..

[19] Piao Z (2023). Adaptive scatter kernel deconvolution modeling for cone-beam CT scatter correction via deep reinforcement learning. Med. Phys..

[20] Yong TH (2021). QCBCT-NET for direct measurement of bone mineral density from quantitative cone-beam CT: A human skull phantom study. Sci. Rep..

[21] Park CS (2023). Validation of bone mineral density measurement using quantitative CBCT image based on deep learning. Sci. Rep..

[22] Kim HJ (2019). A clinical pilot study of jawbone mineral density measured by the newly developed dual-energy cone-beam computed tomography method compared to calibrated multislice computed tomography. Imaging Sci. Dent..

[23] Chang S, Lee SC (2021). A comparative study on the voxel values in alveolar bones acquired by MDCT and newly developed dental dual-energy CBCT. Sensors.

[24] Mallya S (2022). A novel dual-energy cone beam computed tomography device for assessment of jaw bone density. Oral Surg. Oral Med. Oral Pathol. Oral Radiol..

[25] Xu, S. *et al.* Preliminary evaluation of a multi-source CBCT design. *Medical Imaging 2023: Physics of Medical Imaging* 173–181 (SPIE).

[26] Xu S (2023). Evaluation of the feasibility of a multisource CBCT for maxillofacial imaging. Phys. Med. Biol..

[27] Xu S (2023). Volumetric computed tomography with significantly increased quality and accuracy using carbon nanotube x-ray source array. Commun. Eng..

[28] Yin Z, De Man B, Pack J (2009). 3D analytic cone-beam reconstruction for multiaxial CT acquisitions. Int. J. Biomed. Imaging.

[29] Becker AE, Hernandez AM, Schwoebel PR, Boone JM (2020). Cone beam CT multisource configurations: Evaluating image quality, scatter, and dose using phantom imaging and Monte Carlo simulations. Phys. Med. Biol..

[30] Zhang J (2005). Stationary scanning x-ray source based on carbon nanotube field emitters. Appl. Phys. Lett..

[31] Inscoe, C., Lee, Y. Z., Lu, J. & Zhou, O. *Nanostructured Carbon Electron Emitters and Their Applications* 269–288 (Jenny Stanford Publishing, 2022).

[32] Li, B. *et al.* Characterization of a carbon nanotube x-ray source array for a multisource CBCT. *Medical Imaging 2023: Physics of Medical Imaging* 606–611 (SPIE).

[33] van Aarle W (2015). The ASTRA Toolbox: A platform for advanced algorithm development in electron tomography. Ultramicroscopy.

[34] van Aarle W (2016). Fast and flexible X-ray tomography using the ASTRA toolbox. Opt. Express.

[35] Biguri A, Dosanjh M, Hancock S, Soleimani M (2016). TIGRE: A MATLAB-GPU toolbox for CBCT image reconstruction. Biomed. Phys. Eng. Express.

[36] Albus, K. *Phantom Testing: CT (Revised 11–9–2022)*, https://accreditationsupport.acr.org/support/solutions/articles/11000056197-phantom-testing-ct-revised-11-9-2022 (2022).

[37] Fedorov A (2012). 3D Slicer as an image computing platform for the Quantitative Imaging Network. Magn. Reason. Imaging.

[38] Bland JM, Altman DG (1999). Measuring agreement in method comparison studies. Stat. Methods Med. Res..

[39] Gialamas A (2010). Assessing agreement between point of care and laboratory results for lipid testing from a clinical perspective. Clin. Biochem..

[40] Razi T, Emamverdizadeh P, Nilavar N, Razi S (2019). Comparison of the Hounsfield unit in CT scan with the gray level in cone-beam CT. J. Dent. Res. Dent. Clin. Dent. Prospects.

[41] Parsa A (2013). Influence of cone beam CT scanning parameters on grey value measurements at an implant site. Dentomaxillofac. Radiol..

